# How number-space relationships are assessed before formal schooling: A taxonomy proposal

**DOI:** 10.3389/fpsyg.2014.00419

**Published:** 2014-05-14

**Authors:** Katarzyna Patro, Hans-Christoph Nuerk, Ulrike Cress, Maciej Haman

**Affiliations:** ^1^Department of Psychology, University of WarsawWarsaw, Poland; ^2^Knowledge Construction Lab, Knowledge Media Research CenterTübingen, Germany; ^3^Department of Psychology, University of TübingenTübingen, Germany

**Keywords:** infants, number, preschoolers, space, SNA, SNARC, taxonomy

## Abstract

The last years of research on numerical development have provided evidence that spatial-numerical associations (SNA) can be formed independent of formal school training. However, most of these studies used various experimental paradigms that referred to slightly different aspects of number and space processing. This poses a question of whether all SNAs described in the developmental literature can be interpreted as a unitary construct, or whether they are rather examples of different, but related phenomena. Our review aims to provide a starting point for a systematic classification of SNA measures used from infancy to late preschool years, and their underlying representations. We propose to distinguish among four basic SNA categories: (i) cross-dimensional magnitude processing, (ii) associations between spatial and numerical intervals, (iii) associations between cardinalities and spatial directions, (iv) associations between ordinalities and spatial directions. Such systematization allows for identifying similarities and differences between processes and representations that underlie the described measures, and also for assessing the adequacy of using different SNA tasks at different developmental stages.

## Introduction

Over the last 20 years, different experimental paradigms have been employed to examine mental connections between numbers and space. For instance, a parity judgment task with bimanual responses usually reveals the SNARC effect (Spatial-Numerical Associations of Response Codes) which is defined as left-sided response advantage for smaller numbers, and right-sided for larger numbers (Dehaene et al., 1993). This and other kinds of spatial-numerical associations (SNA) were originally thought to be acquired through a long-term experience in a certain cultural environment with specific script direction and formal school training. However, this late development account has been recently challenged because some forms of number-space associations have been discovered to develop before formal schooling.

A closer look reveals that the various SNA investigations in young children have referred to different associations between numbers and space. In fact, the term “space” in number-space relationship is understood and assessed in a variety of ways. Sometimes its directionality is examined (left-right dimension), whereas in other studies its non-directional extension (number lengths). The term “number” has also been used with different connotations: Some studies referred to ordinality (counting) and others to cardinality (numerical magnitudes).

In sum, several theoretical and methodological differences between SNA tasks exist in studies of children, which may address different underlying space and number representations. To date, all SNA studies have been lumped into one category, but no systematic distinction of early SNA measures exists. In the current review, a systematic taxonomy is proposed to enable classifying present and future approaches to research on SNA before school education.

## Distinguishing different SNA effects in preschoolers and infants: toward a taxonomy

While many comprehensive reviews have described varieties of SNAs and their assessments in adults (Gevers and Lammertyn, 2005; Hubbard et al., 2005; Wood et al., 2008), there is only one theoretical article on the pre-linguistic basis of the number-space link (de Hevia et al., 2012), which includes a short overview about developmental studies. While this review is ground-breaking in that it establishes early SNA as a widespread phenomenon, it does not provide a systematization of employed tasks, nor the underlying representations assessed in different age ranges. In the following paragraphs, we emphasize the reasons why a systematization of SNA tasks is important, especially for developmental studies.

First, it is a classical question in psychology, whether different tasks used to assess an underlying construct measure the same construct. The broader the construct is, the more critical this question becomes. The SNA is an example of such a broad construct. Different number representations and their components can be related to space (non-symbolic numerosities, symbolic magnitude, position in a sequence), and different aspects of space can be studied in relation to number processing (length, directionality). In adult studies, not all of these components comprising number-space associations activate the same neurocognitive representations (Turconi et al., 2004; Cohen Kadosh et al., 2007). Therefore, these components should be systematically distinguished.

Second, adult reviews are not sufficient, because different SNAs might emerge at different developmental stages, as different aspects of numerical or spatial information are accessible at different ages. Therefore, general conclusions from adult SNA paradigms may fall short, because the numerical or spatial information employed is inaccessible to young children.

For these reasons, in this review we outline systematic distinctions between SNA effects and their assessment in children before school age. We show that the methods used so far might be based on different aspects of number and space processing—on directional and non-directional space representation, and the representation of cardinal, ordinal or interval numerical information. Because this variety of representations might lead to different ways of defining a number-space link, we suggest distinguishing among at least four main SNA categories, as a first step. Two of them refer to a non-directional number-space mapping: (i) cross-dimensional magnitude processing, and (ii) associations between spatial and numerical intervals. The other two categories refer to directional number-space mapping: (iii) associations between cardinalities and spatial directions, and (iv) associations between ordinalities and spatial directions.

The order of these categories does not reflect the order in which they arise across lifespan. As will be shown later, investigations within some of these categories are focused mostly on one age group and are based on similar experimental paradigms, developed by one or two research groups. Therefore, the current state of research does not allow making strong conclusions about the origins and developmental trajectory of SNA.

Furthermore, we are aware that other distinctions beyond these four categories are possible. For instance, one might distinguish between symbolic and non-symbolic numerosities. However, although symbolic numbers have been employed in some studies reported here, using them as stimuli might be sometimes misleading because only the oldest children master them reliably (see the discussion of the last category). Therefore, we have constrained our categories to those distinctions that have played a major role in prior research on SNA in young children. Thus, our four categories provide a starting point based on the research, but are not meant to preclude additional distinctions that might become relevant in future studies.

Finally, for most of these tasks, it is discussed whether the observed number-space associations are caused by respective representations or rather by corresponding task properties. Although it is beyond a scope of this review to discuss thoroughly all the critics, we will refer to them shortly to provide the reader with a critical overview of the SNA research.

In the next sections, we provide a short outline of assessments and representations tested in each SNA category, the age of the participants with which they have been applied, and describe specific features of the representations and their underlying processes. We also briefly refer to certain controversies aroused by some of these measures. This systematization is summarized in Table 1.

**Table 1:** **The four distinguished SNA categories, their measures, studies in which these measures were used, and mean age of participants**.

**Category of spatial-numerical associations (SNA)**	**Task**^a^****	**Study**	**Mean age (average ages provided as decimal fractions have been recalculated to a “years-months-days” format)**
SNA1: Cross-dimensional magnitude processing	Extraction of an abstract rule across spatial-numerical dimensions	Lourenco and Longo, 2010	*M* = 0 year 9 months 4 days
		de Hevia and Spelke, 2010, Exp. 1	*M* = 0 year 8 months
Space: non-directional Number: cardinal	Numerosity-length matching	de Hevia and Spelke, 2010, Exp. 2, 3	*M* = 0 year 8 months 3 days (Exp. 2),
		de Hevia et al., 2012;	*M* = 0 year 8 months 2 days (Exp. 3)
			*M* = 4 years 1 month
	Line bisection	de Hevia and Spelke, 2009, Exp. 4, 5, 6	*M* = 5 years 1 months (Exp. 4), *M* = 4 years 11 months (Exp. 5), *M* = 4 years 9 months (Exp. 6)
SNA2: Associations between spatial and numerical intervals	Number line task	Siegler and Booth, 2004, Exp. 1, 2	*M* = 5 years 10 months (Exp. 1), *M* = 6 years 1 month (Exp. 2)
		Booth and Siegler, 2006, Exp. 1	*M* = 5 years 8 months
		Ebersbach et al., 2008	*M* = 5 years 3 months
		Berteletti et al., 2010, Exp. 1, 2	*M*_1_ = 4 years 0 month, *M*_2_ = 5 years 0 month, *M*_3_ = 5 years 11 months (Exp. 1)
			*M*_1_ = 4 years 0 month, *M*_2_ = 4 years 11 months, *M*_3_ = 5 years 10 months (Exp. 2)
Space: non-directional^b^ Number: interval		Barth and Paladino, 2011, Exp. 2	*M* = 5 years 7 months
		Fischer et al., 2011	*M* = 5 years 10 months
		Muldoon et al., 2011	*M*_1_ = 4 years 6 months, *M*_2_ = 5 years 2 months
		Slusser et al., 2013, Exp. 1	*M* = 5 years 11 months
		Ebersbach, in press	*M* = 6 years 0 month
SNA3: Associations between cardinalities and spatial directions	Numerosity comparison	Patro and Haman, 2012	*M* = 4 years 0 month
Space: directional Number: cardinal	SNARC tasks	Hoffmann et al., 2013	*M*_1_ = 5 years 6 months, *M*_2_ = 5 years 10 months
		Ebersbach et al., 2013	*M* = 5 years 11 months
SNA4: Associations between ordinalities and spatial directions	Counting	Opfer and Thompson, 2006 (Study1)	*M*_1_ = 3 years 3 months, *M*_2_ = 4 years 0 month, *M*_3_ = 5 years 2 months
		Opfer and Furlong, 2011	*M*_1_ = 4 years 6 months, *M*_2_ = 4 years 6 months, *M*_3_ = 4 years 6 months, *M*_4_ = 4 years 5 months
		Shaki et al., 2012, Exp. 2	*M*_1_ = 4 years 2 months, *M*_2_ = 4 years 1 months, *M*_3_ = 3 years 11 months
Space: directional Number: ordinal	Addition/removal of one object	Opfer and Thompson, 2006 (Study1)	*M*_1_ = 3 years 3 months, *M*_2_ = 4 years 0, *M*_3_ = 5 years 2 months
		Opfer and Furlong, 2011	*M*_1_ = 4 years 6 months, *M*_2_ = 4 years 5 months, *M*_3_ = 4 years 6 months, *M*_4_ = 4 years 5 months
	Spatial search	Opfer et al., 2010, Exp. 1	*M*_1_ = 4 years 6 months, *M*_2_ = 4 years 6 months
		Opfer and Furlong, 2011	*M*_1_ = 4 years 6 months, *M*2 = 4 years 6 months, *M*_3_ = 4 years 6 months, *M*_4_ = 4 years 5 months

a Both ordinal and interval numerical information were studied in relation to space also with a graphic production task (Tversky et al., 1991). Five- and 6-year-old children placed on a paper two stickers representing larger or smaller amounts of objects. These stickers had to be placed in relation to the third, centrally positioned sticker that represented a medium amount of the same things. However, interval relations between numerosities were not mapped by children as spatial distances, and there were no consistent directional biases.

b One recent study (Ebersbach, in press) introduced additional manipulation to the original number-line task by changing the direction of a displayed line (left-to-right vs. right-to-left). Processing of numerical and spatial (non-directional) intervals was still a main representation activated, however, it was affected by another aspect of space processing—its directionality. It shows that our spatial categories are at least in this one case overlapping and future studies might require extention of our taxonomy.

## SNA category 1: cross-dimensional magnitude processing

### Measures

*Extraction of an abstract rule across spatial-numerical dimensions* (de Hevia and Spelke, 2010; Lourenco and Longo, 2010). Eight- and nine-month-old infants were habituated to an abstract rule referring either to numerosity or to length (e.g., color pattern cues assigned to a certain magnitude or ascending/descending orders of magnitude sequences). In a testing phase, a magnitude dimension presented during habituation (e.g., numerosity) was replaced by another dimension (e.g., length). Despite this change, infants detected if an abstract rule, to which they were habituated, had been reversed.

*Numerosity-length matching* (de Hevia and Spelke, 2010; de Hevia et al., 2012). Four-year-old preschoolers were presented with a rule of a positive magnitude matching (larger numerosities paired with longer lines), or an inverse rule (larger-shorter pairings). Most of the children found a correct match in the testing trials, but only in the positive mapping condition. Similarly, following a short familiarization to larger-numerosity-longer-line pairings, 8-month-old infants demonstrated a looking time preference for novel stimuli matched according to the same, recently acquired rule. A familiarization to an inverse rule did not cause any preference in later trials.

*Line bisection* (de Hevia and Spelke, 2009). Five-year-old children were instructed to mark the center of a horizontal line flanked by two arrays of dots with different numerosities. Children placed the midpoint of a line closer to a larger numerosity side.

### Main features of the SNA category 1

The above tasks used different experimental paradigms to explore interrelations between cardinal aspects of non-symbolic numerosities and non-directional spatial dimension - line length. They indicate that length and numerosity are related even for infants and young children, either in a complementary way, carrying together a common abstract rule, or in an interfering way, when a value of one dimension modulates processing of another dimension.

Such kinds of interactions can be explained by theories postulating a generalized system for processing magnitudes of different sorts (Walsh, 2003; Cantlon et al., 2009). Processing of length, number, and other magnitude dimensions share many representational features, for example a continuous and quantitative metric or early ontogenetic and evolutionary beginnings. Therefore, it is postulated that the human mind is predisposed to form a common representational framework for numerical and spatial quantities, or at least to map one dimension onto another. As we will see, this makes the numerosity-length bond unique and qualitatively different from the other SNA categories, for which an influence of cultural factors has been postulated or demonstrated.

These methods are also critically discussed. When stimuli consist of object's collections, certain perceptual set features (density, elements' size) correlated with numerosities may drive participants' choices. For instance, Gebuis and Gevers (2011) challenged the numerical explanation of the line bisection bias. In their experiment, when numerosity was negatively correlated with a set's area, a bias occurred toward a smaller numerosity (larger area). They argued that the bisection bias was visually rather than numerically driven. However, several arguments against their critics were also presented (de Hevia, 2011). Whether children process numerosities independently of perceptual cues becomes now an intensively discussed issue (Cantrell and Smith, 2013; Szűcs et al., 2013; Libertus et al., 2014 for opposite views), which will certainly trigger more studies addressing this question.

## SNA category 2: associations between spatial and numerical intervals

### Measure

*Number-line task* (Siegler and Booth, 2004; Booth and Siegler, 2006; Ebersbach et al., 2008; Berteletti et al., 2010; Barth and Paladino, 2011; Fischer et al., 2011; Muldoon et al., 2011; Ebersbach, in press). Preschoolers (from the age of 4 and older) were instructed to place a target number on a line with 0 or 1 at one end, and 10, 20, 100 or other numbers at the second end (depicted in Arabic or non-symbolic format). Lower accuracy in younger children has been observed, characterized by overestimation of interval distances between smaller numbers (logarithmic scaling). Accuracy in this task increases with age and is correlated with other numerical capabilities.

### Main features of the SNA category 2

This task requires representation of interval scaling instead of cardinal magnitudes. If it is solved correctly, the interval of the numerical magnitude is isomorphic to spatial magnitude of the presented line. Thus, space representation activated here is non-directional (like in SNA1).

A second important feature is that this task enforces spatial mapping of numbers. Thus, its primary purpose should be distinguished from the purposes of SNA1 measures: It does not evaluate whether numbers can be spontaneously related to space, but rather whether such a relationship, if it is induced, is constructed in a systematic interval-scaled way.

The idea that the estimations of the number-line task reflect scaling of a mental number line representation in an isomorphic way (Siegler and Opfer, 2003) has been criticized by various authors. Some authors argue that a logarithmic fit is produced by multi-linear representations for different number ranges (Ebersbach et al., 2008; Moeller et al., 2009). Other suggest that different proportional strategies are applied to solve the task (Barth and Paladino, 2011). The mental processes and strategies underlying the number-space associations in this task are thus still controversial.

## SNA category 3: associations between cardinalities and spatial directions

### Measure

*Numerosity comparison* (Patro and Haman, 2012). Four-years-old children compared numerosities of two bilaterally presented sets. Reactions were faster when a smaller set was on the left side, and a larger one on the right side.

*SNARC tasks* (Ebersbach et al., 2013; Hoffmann et al., 2013). Five-years-old children classified a single number (or set of dots) between two categories (red/green, smaller/larger than x) using left and right buttons. Left-sided reactions were faster to smaller numbers, and right-sided to larger numbers.

### Main features of the SNA category 3

SNA3 and SNA4 differ in a major way from SNA1 and 2, because these associations are about spatial directionality and not about non-directional spatial magnitude. Larger numbers are generally associated with one side of horizontal space (the right side in Western culture), while smaller numbers are associated with the other. In the above tasks, such a directional representation is formed for cardinal numbers.

There are at least two critical issues for this category. First, it is controversial whether an adult-like association is built between numerical and spatial representations (Dehaene et al., 1993), or rather between “smaller/larger” and “left/right” verbal categories (Nuerk et al., 2004; Proctor and Cho, 2006; Gevers et al., 2010). Which kind of association preschoolers build, has not yet been examined. Second, reading direction was traditionally believed to be a main source of SNARC (Zebian, 2005; Shaki and Fischer, 2008), whereas other not determined pre-literate factors may build SNA in preschoolers. If such factors are among early acquired cultural skills, it would strengthen the difference between SNA1 and SNA3 because the first category may be more innately determined.

## SNA category 4: associations between ordinalities and spatial directions

### Measures

*Counting* (Opfer and Thompson, 2006; Opfer and Furlong, 2011; Shaki et al., 2012). English-, Hebrew-, and Arabic-speaking preschoolers (age 3–6) were asked to count objects aligned horizontally in a row. Most of English-speaking children counted from left to right, whereas Hebrew- and Arabic-speaking children counted from right to left.

*Addition/removal of one object* (Opfer and Thompson, 2006; Opfer and Furlong, 2011). In an addition task, English-speaking preschoolers (age 3–5) were presented with three objects aligned in a row, and asked to add one object. In a subtraction task, they were presented with four objects and asked to remove one of them. The number of participants who added to and removed from the correct right end increased with age, but it was still below 50%.

*Spatial search* (Opfer et al., 2010; Opfer and Furlong, 2011). Four-year-old English-speaking children were presented with two boxes containing seven compartments, numbered in a left-to-right or right-to-left order. An experimenter showed a hidden star in one compartment of the first box, and asked children to find a similar star in the second box, in the compartment with the same number. Children performed more accurately in a condition when compartments were numbered from left to right than in a condition with the reversed numerical order.

### Main features of the SNA category 4

The tasks from this category are based on directional processing of numerical orders. Ordering tasks may be subdivided into two categories: One category without necessary access to magnitude (e.g., counting tasks) and another category, in which order necessarily involves magnitude access or cardinality. For instance, ascending/descending numerosity-length matching (de Hevia and Spelke, 2010) requires some sort of order representation which is based on magnitude (smaller comes before larger). Therefore, in this category, we suggest to include only those studies in which a position of a number in a sequence (four comes before five, rightmost element should be removed), and not its magnitude, is relevant for solving a task.

Research with adults suggests that ordinal and cardinal processing of numbers rely at least partly on different processes. For instance, neural activation observed during classification of numbers as smaller or larger than a target has a different spatio-temporal pattern than during classifying them as coming before or after a target (Turconi et al., 2004). There is a strong agreement that both kinds of numerical processes constitute a number semantics (Sury and Rubinsten, 2012), but their contributions to building a numerical representation, especially in children, might be different.

In young preschoolers, the fixed order of a counting list is not primarily a numerical representation. Access to numerical semantics through symbolic notation establishes itself through the whole preschool period (Wynn, 1990; Le Corre and Carey, 2007). Although 3-year-olds might recite sequences of number words, they are usually not yet able to assign all learned numerals to corresponding values or to use a counting procedure to establish a set numerosity.

These considerations make the conceptual distinction between cardinal tasks (SNA3) and ordinal tasks (SNA4) valid, but a distinction does not imply that ordinal and cardinal knowledge are unrelated—the fixed order of a counting list may provide a scaffold for later understanding of cardinality. According to the cardinal principle of counting, the magnitude of the last ordinal element equals the cardinality. Children realize that quite late, usually only around the age of four. Once children have realized the relation between symbolic number order and number cardinality, however, order tasks like a counting or digit search task may become theoretically ambiguous, because it is sometimes not entirely clear to what extent ordinal and cardinal aspects of numbers contribute to the observed spatial associations.

Another important feature of SNA4 refers to its validity. In the tasks from this category, object stimuli are already arranged spatially forming a row. Thus, alike in SNA2, and contrary to SNA1 and 3, this task induces spatial coding and cannot evidence spontaneous number-space mapping. Note, however, that only a spatial location of response is enforced, but not directionality, because children can also count or subtract from the middle.

## Summary

In the taxonomy proposed here, we have distinguished four basic categories of SNA research in children before school age. Some of them share basic features, like directional components, or using a spatial magnitude as a basic mapping metric, but they differ at the same time in other aspects (processing ordinal, cardinal or interval number magnitudes).

These four distinguished SNA categories, although based on different number and space representations, are not intended to be exclusive. Our aim was to accentuate that although they are overlapping and related to each other, their basic mechanisms, developmental trajectory, and susceptibility to cultural experience might be different.

These categories are a starting point for categorizing the most important distinctions in current preschool SNA research. They may not be comprehensive for all future results. For instance, we have outlined that the distinction between symbolic and non-symbolic magnitudes is not of major importance in prior developmental studies on SNA, but it is possible that future studies will bring new insights about the symbol-grounding problem that will require extension of this taxonomy.

Developmental studies on SNA are still sparse and other research paradigms might bring new insights into individual trajectories, new distinctions, and the causal relations of different number-space associations. They could also help to clarify the issues of validity of different SNA measures. However, we believe that the current taxonomy helps to understand that the growing evidence about SNA before schooling is not unitary, and should be categorized with consideration of the major distinctions of underlying space and number concepts used in empirical studies to date.

### Conflict of interest statement

The authors declare that the research was conducted in the absence of any commercial or financial relationships that could be construed as a potential conflict of interest.
